# *NTRK* Gene Fusions in Non-Small-Cell Lung Cancer: Real-World Screening Data of 1068 Unselected Patients

**DOI:** 10.3390/cancers15112966

**Published:** 2023-05-29

**Authors:** Tobias Raphael Overbeck, Annika Reiffert, Katja Schmitz, Achim Rittmeyer, Wolfgang Körber, Sara Hugo, Juliane Schnalke, Laura Lukat, Tabea Hugo, Marc Hinterthaner, Kirsten Reuter-Jessen, Hans-Ulrich Schildhaus

**Affiliations:** 1 Department of Hematology and Medical Oncology, University Medical Center Göttingen, 37075 Göttingen, Germany; 2 Göttingen Comprehensive Cancer Center (G-CCC), Lungentumorzentrum Universität Göttingen, 37075 Göttingen, Germany; 3 Institute of Pathology, University Medical Center Göttingen, 37075 Göttingen, Germany; 4 Tyrolpath Obrist Brunhuber GmbH and Krankenhaus St. Vinzenz, 6511 Zams, Austria; 5 Lungenfachklinik Immenhausen, 34376 Immenhausen, Germany; 6 Department of Pneumology Evangelisches Krankenhaus Weende, 37075 Göttingen, Germany; 7 Discovery Life Sciences, 34119 Kassel, Germany; 8 Department of Heart, Thoracic and Vascular Surgery, University Medical Center Göttingen, 37075 Göttingen, Germany

**Keywords:** NSCLC, lung cancer, *NTRK*, targeted therapy, immunohistochemistry, fluorescence in situ hybridization, FISH, RNA-based next-generation sequencing

## Abstract

**Simple Summary:**

The identification of potential molecular alterations is standard in the diagnostic pathway of non-small-cell lung cancer (NSCLC). The aim of this study is to determine the prevalence of *NTRK* fusions in NSCLC in a routine diagnostic setting using immunohistochemistry, fluorescence in situ hybridization, and RNA-based next-generation sequencing. A total of 1068 unselected consecutive patients with NSCLC were screened in two scenarios, either with initial IHC followed by RNA-NGS (*n* = 973) or direct FISH testing (*n* = 95). In total, 0.2% of all patients were *NTRK* positive. Both RNA-NGS and FISH are suitable to determine clinically relevant *NTRK* fusions in a real-world setting. RNA-NGS or FISH *NTRK* positive results were mutually exclusive with alterations in *EGFR/ALK/ROS1/BRAF/RET* or *KRAS*.

**Abstract:**

(1) Background: The main objectives of our study are (i) to determine the prevalence of *NTRK* (*neurotrophic tyrosine kinase*) fusions in a routine diagnostic setting in NSCLC (non-small cell lung cancer) and (ii) to investigate the feasibility of screening approaches including immunohistochemistry (IHC) as a first-line test accompanied by fluorescence in situ hybridization (FISH) and RNA-(ribonucleic acid-)based next-generation sequencing (RNA-NGS). (2) Methods: A total of 1068 unselected consecutive patients with NSCLC were screened in two scenarios, either with initial IHC followed by RNA-NGS (*n* = 973) or direct FISH testing (*n* = 95). (3) Results: One hundred and thirty-three patients (14.8%) were IHC positive; consecutive RNA-NGS testing revealed two patients (0.2%) with *NTRK* fusions (*NTRK1-EPS15* (*epidermal growth factor receptor pathway substrate 15*) and *NTRK1-SQSTM1* (*sequestosome 1*)). Positive RNA-NGS was confirmed by FISH, and *NTRK*-positive patients benefited from targeted treatment. All patients with direct FISH testing were negative. RNA-NGS- or FISH-positive results were mutually exclusive with alterations in *EGFR* (*epidermal growth factor receptor*), *ALK* (*anaplastic lymphoma kinase*), *ROS1* (*ROS proto-oncogene 1*), *BRAF* (*proto-oncogene B-Raf*), *RET* (*rearranged during transfection*) or *KRAS* (*kirsten rat sarcoma viral oncogene*). Excluding patients with one of these alterations raised the prevalence of *NTRK*-fusion positivity among panTrk-(tropomyosin receptor kinase-) IHC positive samples to 30.5%. (4) Conclusions: *NTRK* fusion-positive lung cancers are exceedingly rare and account for less than 1% of patients in unselected all-comer populations. Both RNA-NGS and FISH are suitable to determine clinically relevant *NTRK* fusions in a real-world setting. We suggest including panTrk-IHC in a diagnostic workflow followed by RNA-NGS. Excluding patients with concurrent molecular alterations to *EGFR/ALK/ROS1/BRAF/RET* or *KRAS* might narrow the target population.

## 1. Introduction

The Trk family of neurotrophic tyrosine kinases, also known as tropomyosin receptor kinases, consists of three proteins, TrkA, TrkB, and TrkC. These proteins are encoded by three genes, *NTRK1* on chromosome 1q21-22, *NTRK2* on 9q22.1, and *NTRK3* on 15q25, respectively [1]. The proteins share structural and functional similarities and are composed of extracellular domains, including IgG-like parts, a transmembrane domain, and a catalytically active intracellular tyrosine kinase domain [1,2,3]. Splice variants that modify protein structure and function and create isoforms have been described for all three genes. Trk proteins are physiologically activated by ligands such as nerve growth factor (NGF), brain-derived neurotrophic factor (BDNF), and neurotrophin-3 (NT-3, also NT-4/5) [4]. As regulators of tissue development during embryogenesis and maintenance of function in adult tissue, Trk kinases are expressed in normal central and peripheral nerve tissue as well as in beta cells of the endocrine pancreas, monocytes, and bone tissue.

Recently, oncogenic translocations involving *NTRK1*, *NTRK2*, and *NTRK3* genes have been described [5,6,7,8,9,10,11,12,13,14,15,16,17,18,19,20,21,22,23,24,25,26,27,28,29,30,31,32,33,34,35,36,37,38,39,40]; for a compiled overview of the literature of previously published *NTRK* fusions in NSCLC, including differentiation by histology, see Table 1.

Biologically, most of these fusions result in a chimeric transcript in which regulatory parts, i.e., extracellular and transmembrane domains, are replaced by parts of another gene that facilitate ligand-independent autonomous dimerization and activation, and thus, the oncogenic transformation of cells. Unsurprisingly, these changes have been found among malignancies of the central nervous system, e.g., adult- and pediatric-type gliomas and glioblastomas [18]. However, a wide variety of non-CNS (central nervous system) tumors turned out to harbor *NTRK* fusions as well, among them carcinomas of the lung, colorectal cancers, thyroid cancer, and cholangiocellular carcinomas. Exceptionally rare tumors, e.g., secretory carcinomas of the breast and their counterparts in salivary glands, infantile fibrosarcomas, and congenital mesonephric blastomas, are molecularly defined by *NTRK* fusions. These tumors harbor *NTRK* fusions in nearly 100% of cases, predominantly the *NTRK3*-*ETV6* variant. Another rare group of tumors, including spitzoid melanomas, papillary thyroid cancer, and inflammatory myofibroblastic tumors, seems to show a moderate frequency of *NTRK* fusions. However, more prevalent cancers, among them non-small-cell lung cancer, harbor *NTRK* fusions very infrequently. Data from clinical registers and clinical trials indicate a prevalence of *NTRK* fusions among lung cancers below 1% [19,22,26,30,31,35,41,42,43]. Population-based data from a clinical all-comer series of Caucasian lung cancer patients are not yet available.

Nevertheless, clinical trials have demonstrated impressive responses of *NTRK*-fused cancers to two tyrosine kinase inhibitors with anti-Trk activity, Larotrectinib and Entrectinib [44,45]. Larotrectinib and Entrectinib were recently approved by the U.S. Food and Drug Administration (FDA) and the European Medicines Agency (EMA) for the treatment of *NTRK*-positive tumors irrespective of specific tumor entity [46,47,48,49].

Given the high clinical benefit of Trk inhibitors and the very low prevalence of *NTRK* fusions among major groups of human cancers, it is extremely important to define a reasonable screening strategy that, on one hand, safely detects *NTRK*-fusion-positive patients with high sensitivity and specificity and, on the other hand, takes into consideration additional important factors, such as cost-effectiveness, time to diagnosis, and testing feasibility due to tissue characteristics. Basically, several methods are available to detect *NTRK* fusions: (i) DNA-(desoxyribonucleic acid-) based (hybrid capture) next-generation (NGS) assays, (ii) RNA-based NGS approaches, either target enrichment or hybrid capture methods, (iii) fluorescence in situ hybridization (FISH), and (iv) immunohistochemistry (IHC) [19,50,51]. DNA- and RNA-based sequencing and FISH have been utilized in clinical trials, whereas IHC has been proposed as a pre-screening test [44,45,51]. It has been shown, however, that DNA-based hybrid capture approaches suffer from severe limitations since some specific fusion variants could not be detected by this technology [14]. Therefore, RNA-based NGS is currently considered a gold standard or reference method and is recommended as the primary test approach if available [52]. In contrast, these assays are not always available and extremely demanding in terms of costs, time, and tissue/RNA quality. Therefore, there is an urgent clinical need to develop reasonable screening strategies for major cancer types, such as lung cancer.

In this study, we aimed—as the primary objective—to determine the prevalence of *NTRK* gene fusions in an unselected series of consecutive lung cancer patients. The secondary objective was to determine the overall feasibility of *NTRK* screening approaches in a routine diagnostic setting. Furthermore, additional goals were (a) to describe the specificity and potential diagnostic pitfalls of immunohistochemistry as a potential first-level pre-screening approach, (b) to compare the performance and results of IHC, FISH, and RNA-based sequencing as diagnostic techniques, and (c) to describe clinical and histologic phenotypes of *NTRK* fusion-positive patients.

## 2. Materials and Methods

### 2.1. Patients and Samples

**Figure 1:**
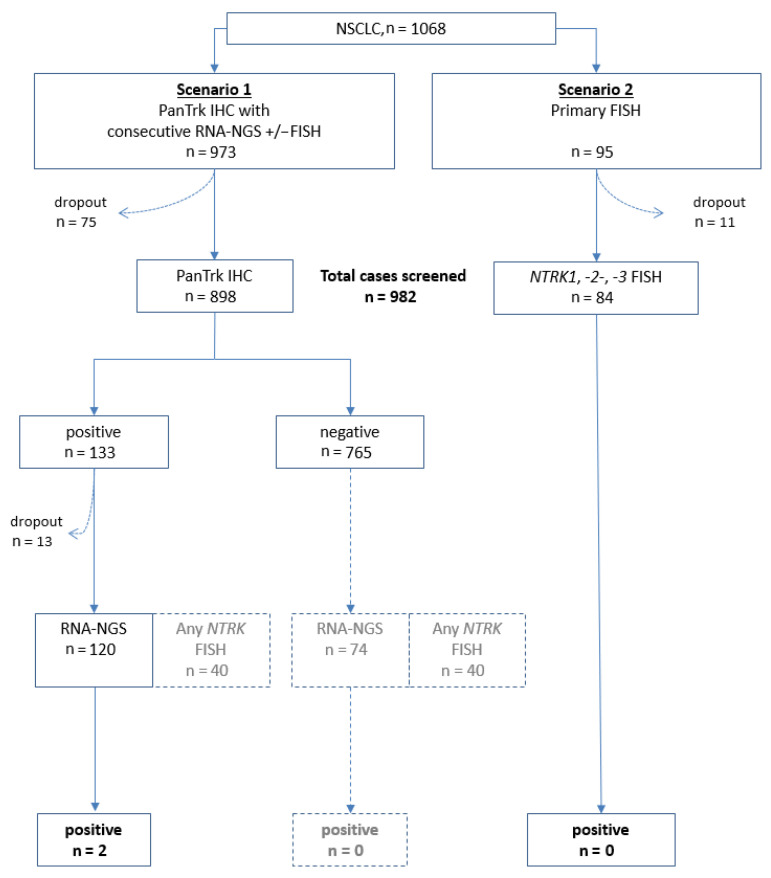
Testing algorithm of this study. A total of 1068 cases were intended to be tested. In total, 982 samples underwent prospective screening either with IHC followed by NGS or with FISH (scenarios 1 and 2). A subset of samples with sufficient residual tumor tissue was retrospectively cross-tested with other methods (grayed-out boxes).

During an 18 month period, 1068 unselected consecutive NSCLC patients were prospectively included in a screening for *NTRK* fusions as part of our routine diagnostic procedures at the Lung Cancer Center Göttingen (Lungentumorzentrum Universität Göttingen as part of the Göttingen Comprehensive Cancer Center, G-CCC). Patients were included irrespective of tumor stage at the time of diagnosis and independent of tumor histology and molecular subtype. Tumor samples were randomly tested either by IHC and subsequent RNA-based next-generation sequencing with FISH confirmation (Figure 1, Scenario 1) or by direct FISH testing (Figure 1, Scenario 2). After a dropout of 86 cases due to limited material, 982 samples underwent complete prospective screening either with IHC followed by NGS (scenario 1, *n* = 898) or with FISH (scenario 2, *n* = 84). A subset of IHC-positive samples of Scenario 1 with sufficient residual tumor tissue was retrospectively cross-tested by FISH (*n* = 40). A total of 13 IHC-positive cases were not suitable for RNA-NGS analysis due to failure or lack of tumor material or technical errors. In total, 120 IHC-positive cases were examined by RNA-NGS. As part of cross-testing, an additional 74 IHC-negative cases were examined by RNA-NGS. Furthermore, a subset of IHC-negative samples was tested by FISH as part of cross-testing (*n* = 40; see Figure 1 and Table 2 and Table 3 for details). The molecular diagnostic algorithm for *KRAS, EGFR, BRAF* and *ALK, ROS1*, and *RET* followed the institutional approach as previously described. In brief, mutational analysis was performed by DNA-NGS and the detection of translocations by FISH or RNA assay [53].

Of 982 samples with complete molecular analysis, 523 samples were mediastinal, endobronchial, or transthoracic biopsies of primary tumors, 290 were surgical resections (among them, 189 primary tumors and 101 metastases), 9 were cell blocks of malignant effusions, 143 samples were biopsies from metastases, and 7 samples were from recurrent disease (*n* = 10 samples from unknown origin). Cytology specimens, i.e., smears or cytospin preparations, were excluded, as were lung metastases of extra-pulmonary primaries and small-cell lung cancers. NSCLC subtypes were established based on the current WHO (world health organization) and IASLC (International Association for the Study of Lung Cancer) classification [54,55] by an experienced lung cancer pathologist (H.-U.S.). Molecular subtyping and PD-L1 testing were carried out as part of routine procedures as previously described [56,57,58,59]. *NTRK*-fusion-positive patients were enrolled and treated in clinical trials after central confirmation.

### 2.2. Immunohistochemistry

Slides of 3–4 µm thickness were cut from paraffin blocks and heat-induced epitope retrieval was carried out at pH 9.0 by using the Envision Flex + high pH System at a DAKO Autostainer Link platform (DAKO, Agilent, Santa Clara, CA, USA). Monoclonal rabbit panTrk antibody C17F1 (Cell Signaling Technology) was used (at a 1:25 dilution; 30 min incubation time). DAB served as chromogen. Tissue from cancers with known *NTRK* fusion status, e.g., secretory breast carcinoma, and brain tissue were used as controls for establishing and validation of staining; brain tissue was used as on-slide controls. Cases were considered “IHC positive” if any staining at any intensity occurred (any staining above background, visible at 200× or 400× magnification, irrespective of sub-cellular location of staining within the tumor, including membranous, cytoplasmic, and nuclear staining) during the screening phase of this project. IHC slides were evaluated by one dedicated pathologist (H.-U.S.).

### 2.3. RNA-Based Sequencing

Total nucleic acid isolation was performed with the Agencourt FormaPure Kit (Beckman Coulter, Brea, CA, USA), according to the manufacturer’s protocol. RNA was quantified with the Qubit hsRNA Assay and 200 ng were used for subsequent sequencing library preparation with an Anchored Multiplex polymerase chain reaction (AMP; Archer Fusionplex CTL panel). For library sequencing an Illumina MiSeq device (MiSeq Control Software Version 2.6.2.1) was used and data were analyzed with the Archer Analysis bioinformatics platform 4.1. Only fusion variants that were labeled as “strong evidence” were considered *NTRK* fusion-positive. Evaluation of sequencing data was conducted by a dedicated molecular biologist (K.R.-J.).

### 2.4. NTRK FISH

Fluorescence in situ hybridization for *NTRK1*, *NTRK2*, and *NTRK3* translocations was carried out by using the ZytoLight SPEC NTRK1 Dual Color Break Apart Probe, ZytoLight SPEC NTRK2 Dual Color Break Apart Probe, and ZytoLight SPEC NTRK3 Dual Color Break Apart Probe, respectively (all from Zytovision, Bremerhaven, Germany). Hybridization was done as previously described [53,60]. One hundred consecutive tumor cell nuclei were evaluated and the percentage of break apart and/or isolated orange signals was noted. Cases were considered positive if at least 20% of nuclei showed one of these aberrant signal patterns. FISH assays were evaluated by dedicated pathologists with specific experience in ISH evaluation (K.S., H.-U.S.; additional evaluations for retrospective cross-method validations were carried out by Ann.R.).

### 2.5. Statistics

SPSS software (IBM Corp., IBM SPSS Statistics for Windows, Version 25.0. Armonk, NY, USA) was used for descriptive statistical analysis.

## 3. Results

### 3.1. Feasibility of Applied Screening Approaches and Prevalence of NTRK Fusions

A total of 1068 unselected consecutive patients with NSCLC was screened in two scenarios, either with primary initial IHC followed by RNA-NGS (*n* = 973, Scenario 1) or primary direct FISH testing (*n* = 95, Scenario 2). Overall, 982 out of 1068 samples could be successfully evaluated (91.9%). The drop-out rate was 11/95 (11.6%) for FISH (Scenario 2), and 75/973 (7.7%) for IHC (Scenario 1). Additionally, 13/133 (9.8%) of IHC-positive samples could not be analyzed by NGS (as part of Scenario 1). The reason for screening failures was the overall amount of available tumor tissue or harsh decalcification. In Scenario 1, 133/898 (14.8%) samples were IHC-positive. A total of 2 out of 120 (1.7%) evaluable IHC-positive samples showed an NTRK fusion using RNA-NGS. In Scenario 2, no NTRK-positive samples were detected. Furthermore, cross-testing (including RNA-NGS of IHC-negative samples and FISH testing of samples from Scenario 1) did not reveal any additional NTRK fusion-positive cases. The overall prevalence of NTRK fusion was 0.2% (2/982). Patients’ characteristics, diagnoses, and molecular findings are displayed in Table 2 and Table 3.

All morphologic subtypes, except adenocarcinomas, were negative for *NTRK* fusions. The two patients with positive *NTRK* fusion showed no co-existing driver alteration concerning *EGFR*, *BRAF*, *ALK*, *ROS1*, *RET*, or *KRAS*.

### 3.2. Characteristics of NTRK-Fusion-Positive Patients

**Figure 2:**
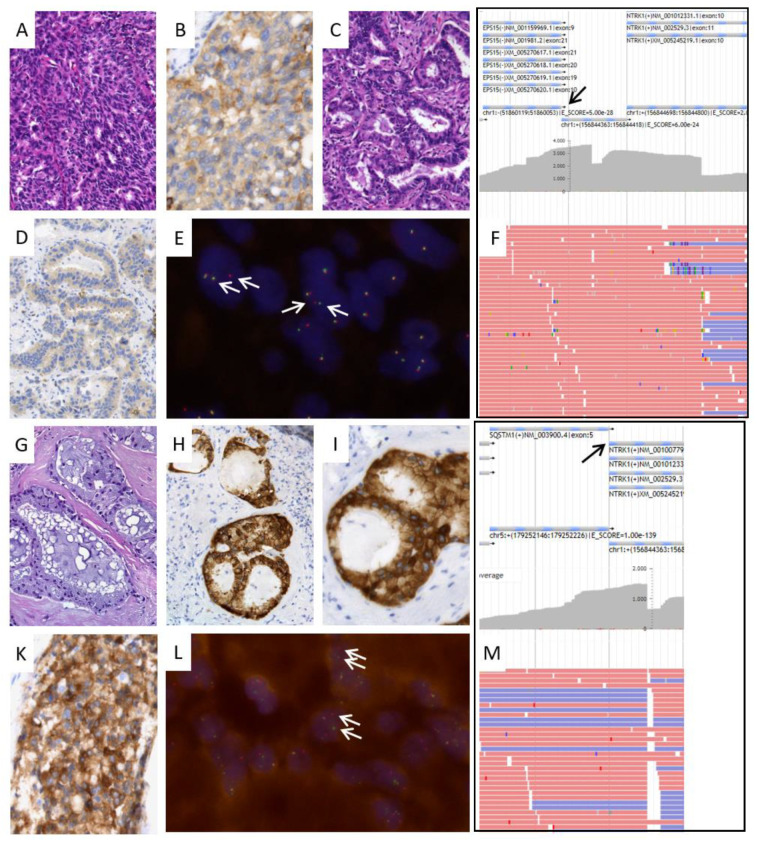
Morphologic and molecular characteristics of *NTRK* fusion-positive lung cancer cases. Case 1: *EPS15-NTRK1* fusion. (**A**,**C**): This adenocarcinoma shows solid and acinar morphology (hematoxylin-eosin staining, specimen from wedge resection). (**B**,**D**): Moderate to strong immunohistochemical staining of tumor cell membranes and cytoplasm with panTrk antibody in solid tumor aspect of the primary tumor; weak staining in acinar areas. (**E**): Fluorescence in situ hybridization (FISH) analysis showing multiple break apart signals (arrows), indicating *NTRK1* gene translocation. (**F**): RNA-based NGS analysis showing multiple reads of an *EPS15-NTRK1* fusion gene at the fusion site (arrow), confirming translocation of NTRK1 gene. Case 2: *SQSTM1-NTRK1* fusion, (**G**): Mucinous morphology in a lymph node metastasis (hematoxylin-eosin staining). (**H**,**I**): Immunohistochemical staining with panTrk antibody demonstrating strong membranous staining. (**K**): Immunohistochemical staining in another lymph node metastasis confirming homogeneous strong staining. (**L**): Fluorescence in situ hybridization (FISH) analysis showing multiple break apart signals (arrows), indicating *NTRK1* gene translocation. (**M**): RNA-based NGS analysis showing multiple reads of an *SQSTM1-NTRK1* fusion gene at the fusion site (arrow), confirming translocation of *NTRK1* gene. Original magnifications ×100 (**A**), ×200 (**C**,**D**,**G**,**H**), ×400 (**B**,**I**,**K**), ×630 (**E**,**I**).

We describe two subtypes of *NTRK* fusions in NSCLC, one *EPS15* (exon 9)—*NTRK1* (exon 10) and one *SQSTM1* (exon 6)—*NTRK1* (exon 10) fusion. Both genes have previously been described as fusion partners for *NTRK* in NSCLC. *EPS15*, which encodes the epidermal growth factor receptor substrate 15, has been described as fusing with *NTRK1*, but with exon 21 of *EPS15* as the fusion site [19,32]. Sequestosome-1 (*SQSTM1*) is known as a translocation partner of *NTRK1, -2,* and *-3* with breakpoints located in exon 5 or 6 of *SQSTM1* [33,34,35]. Based on histology, both *NTRK*-positive cases represent moderately differentiated adenocarcinomas with a predominant acinar morphology. Additional mucinous or solid components occurred (Figure 2). Both cases were negative for *KRAS*, *BRAF*, and *EGFR* mutations, *ALK*, *ROS1*, and *RET* fusions, as well as for *MET* amplification.

**Figure 3:**
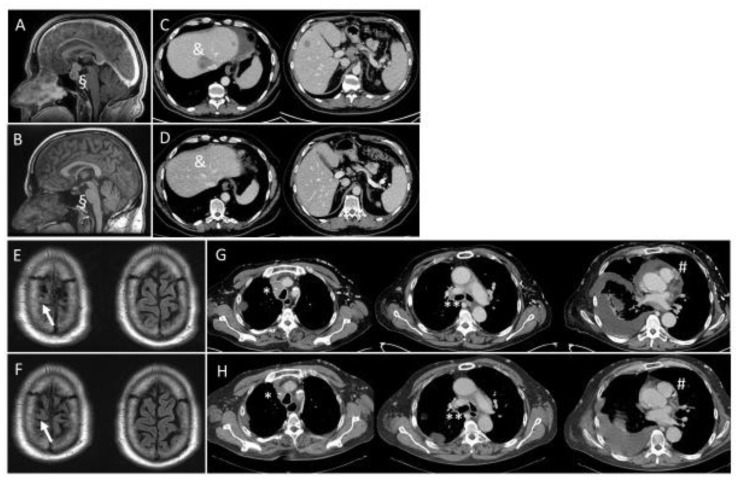
MRI and CT scans of two patients with *NTRK* fusion and Trk-directed therapy. Patient 1 (**A**–**D**) and patient 2 (**E**–**H**) showed responses to Trk-targeted therapy. (**A**,**C**,**E**,**G**) show MRI and CT scans at baseline. (**B**,**D**) show follow-up at month 27 (patient 1) and (**F**,**H**) at month 6 (patient 2). Metastatic sites, (**A**–**D**): § sellar/suprasellar, & liver parenchyma, (**E**–**H**): ↑ right precentral gyrus, * mediastinal, ** hilar, # pericardial effusion and cardiac metastasis.

The two patients are male with a comparably long-standing history of their disease and primary diagnosis of lung cancer at younger ages (patient 1 at the age of 51 with oligometastatic disease and patient 2 at the age of 45 with stage IIIA disease). *NTRK* diagnostic was initiated within progressive and stage IV disease after surgery, radiotherapy, and systemic therapy. Patient 1 is a former smoker with a history of 10 pack years and patient 2 is a current smoker with 45 pack years. Both patients were included in clinical trials with Trk-targeted therapy, patient 1 within 27 months after primary diagnosis of lung cancer and patient 2 15 years and 9 months following the primary diagnosis. Patient 1 received Trk-directed therapy as a second-line treatment for stage IV disease and patient 2 as first line systemic therapy for stage IV disease. Both patients responded with partial remission. After the initiation of Trk-targeted therapy the clinical performance status improved to a normal level in both cases. Patient 1 is still undergoing Trk-targeted therapy at month 45 without recurrent disease. Patient 2 died of an exacerbation of underlying chronic obstructive lung disease, having been treated with Trk-targeted therapy without recurrence for 42 months. Figure 3 illustrates the intra- and extracranial responses in both patients documented by MRI (magnetic resonance imaging) and CT (computerized tomography) scans.

### 3.3. Positive Predictive Value and Diagnostic Characteristics of Immunohistochemistry

Our study represents one of the largest series of Trk IHC in NSCLC with roughly 1000 samples. Based on our experience, we noticed that the positive predictive value was low with only 2 out of 133 IHC positive samples where an *NTRK* fusion could be detected by subsequent sequencing. The *NTRK*-positive tumors showed weak to moderate cytoplasmic and membranous staining with varying intensities between different tumor manifestations (Figure 2).

We did not notice any major difference in IHC staining patterns between both fusion subtypes. Nuclear staining was not observed in these *NTRK1*-fused cancers.

**Figure 4:**
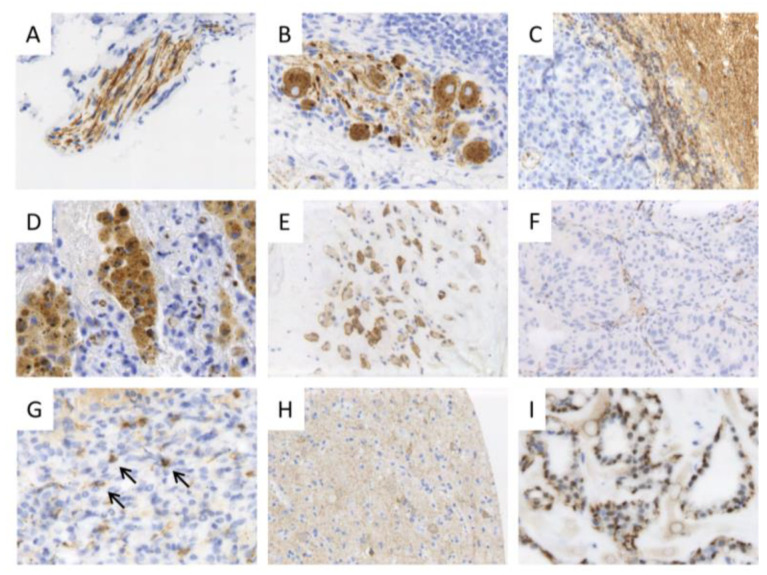
Pitfalls in evaluating panTrk IHC staining. Trk IHC in non-neoplastic tissue, controls, and *NTRK* fusion-negative tumors. Trk proteins are physiologically expressed in structures of the peripheral and central nervous system, such as nerval fibers (**A**), ganglion cells (**B**), and brain tissue ((**C**)—right; Trk IHC-negative metastasis of an NSCLC on the left side). Furthermore, macrophages (**D**), reactively changed muscle fibers (**E**), and some vessels (**F**) can demonstrate a Trk expression. (**G**): Inflammatory cells surrounding a tumor may show positivity (arrows). (**H**): Brain tissue with weak staining was used as on-slide control. (**I**): Secretory breast cancer tissue with proven *NTRK3-ETV6* fusion can be used as control. Tumor cells show weak cytoplasmic and moderate to strong nuclear staining. Original magnifications ×100 (**E**,**H**), ×200 (**A**–**D**,**F**,**G**,**I**).

Overall, Trk staining was weak in tumor tissue. We failed to detect a correlation between staining intensity and *NTRK* fusion since some of the cases with the strongest staining turned out to be fusion negative. Moreover, Trk staining in fusion-positive samples was at least focally remarkably weak. We observed a reproducible and uniform Trk expression in some neoplastic and non-neoplastic tissues, which could be used as controls for IHC, such as secretory breast cancer or brain tissue. Tumor-infiltrating inflammatory cells, structures of peripheral nerval tissue. Or reactively changed skeletal muscle can express Trk at high levels, and therefore represent potential pitfalls for Trk IHC evaluation (Figure 4).

Since panTrk immunohistochemistry does not discriminate between wild-type and fused Trk proteins, it is not surprising that carcinomas can express these proteins as well. The expression levels were the highest among sarcomatoid carcinomas, poorly differentiated NOS carcinomas, and neuroendocrine and squamous cell carcinomas. Staining in these tumors could be both membranous and cytoplasmic; nuclear staining was not observed. Based on our experience within this screening project and with other tumor tissues and previous reports, nuclear Trk staining is probably specific to *NTRK3*-fused cancers [17]. As mentioned above, we retrospectively examined a number of IHC-negative samples in the context of a cross-method validation by RNA-NGS and/or FISH, but did not observe any IHC-negative/RNA-NGS-positive cases.

### 3.4. Comparison of Molecular Test Approaches

We further examined NGS-negative cases with positive Trk expression based on IHC with FISH but could not identify additional positive samples. However, RNA-NGS-positive tumors were also clearly positive with *NTRK1* FISH. We did not observe any *NTRK2*- or *NTRK3*-positive FISH sample. Therefore, we saw a 100% concordance between FISH and RNA-NGS based on the limited number of samples that were analyzed with both methods.

We obtained valid FISH results within two working days, whereas the entire RNA-NGS workflow, including RNA extraction and quantification, library preparation, sequencing, and bioinformatics, took more than 10 working days in our hands.

## 4. Discussion

Our major goal was to determine the prevalence of *NTRK*-fused lung cancers under real-world conditions. Based on more than 1000 patients, we detected a frequency of 0.2% in a prospective series of unbiased and unselected lung cancer patients. This prevalence falls within the range of previous reports from clinical trials and more specific *NTRK* screening programs [19,22,26,30,31,35,41,42,43]. Thus, our study does not only confirm the previously published prevalence but also demonstrates that *NTRK*-fused NSCLC can be detected by an approach of a two-step screening consisting of IHC as a pre-screening method and subsequent RNA-based NGS of IHC-positive samples. Monitoring the prevalence of *NTRK*-fusion-positive samples may be used as a validation tool of screening workflows in other institutions as well. Our data indicate that a 0.2% positivity rate in an all-comer population can serve as a reasonable target value.

We demonstrate that *NTRK* screening is feasible in a routine diagnostic setting as well. The drop-out rate in our proposed workflow of IHC followed by RNA-NGS was 9.0% (as opposed to 11.6% in a FISH-only approach), and we conclude that *NTRK* testing can be performed on specimens from resections, biopsies, and cell blocks if the tumor tissue fulfills basic requirements for IHC and molecular tests.

In our study, we used IHC as a pre-screening test. Based on our experience with nearly 1000 immunohistochemical samples, we describe a comparably low positive predictive value of IHC: only 1.5% of all IHC-positive NSCLC samples turned out to harbor a *NTRK* fusion. This is mainly due to the fact that IHC cannot discriminate between the expression of wild-type Trk proteins and chimeric fusion proteins. However, prevalence was higher among adenocarcinomas (2/701 = 0.29%). Among 568 non-squamous NSCLC with available simultaneous testing results of *EGFR* and *KRAS*, 297 were wild-type for both genes. In this group, the prevalence for *NTRK* positivity was 2/297 = 0.67%. Simultaneous testing for *EGFR/KRAS/BRAF/ALK/ROS1/RET* was available for 339 patients with NSCLC, with negative results for all genes in 175 cases. Among the latter group, the prevalence of *NTRK* rearrangements was 1.14%. We did not observe any *NTRK* fusions in tumors other than adenocarcinomas. This is in line with previous reports where the vast majority of *NTRK*-positive cancers were adenocarcinomas as well. Neuroendocrine carcinomas and squamous carcinomas are infrequently involved [13,15,31,33,34,37].

Based on our experience with Trk IHC, we describe potential diagnostic pitfalls and reasonable control tissues (Figure 4). In our study, we regarded RNA-based NGS as a gold standard and reference method. However, FISH analyses were completely concordant with NGS. Although we could only investigate a relatively small subset of our samples with both methods, we did not find any discrepant cases. Therefore, we conclude that FISH is a valid and reliable method to detect *NTRK* fusions as well. A major advantage of this technology is the comparably short time to diagnosis, with a disadvantage being the comparably higher number of slides cut from tumor blocks needed to examine all three *NTRK* genes.

Given the extremely low frequency of *NTRK* fusions among lung cancer patients on one hand and the significant clinical benefit of Trk inhibitor treatment in *NTRK*-fusion-positive patients on the other hand, it is extremely important to establish a diagnostic algorithm that is sensitive and specific, tissue-sparing, and cost- and time-efficient and that allows reliable testing in a routine environment.

In this report, we demonstrate that a diagnostic workflow, including IHC and RNA-based sequencing, has the capability to detect *NTRK*-fusion-positive samples under routine conditions. We identified positive patients at the expected frequency. *NTRK* fusion positivity could be centrally confirmed as part of a clinical trial, and patients responded to anti-Trk treatment. These clinical data provide further evidence that our workflow was reliable and valid.

It has been demonstrated that RNA-based sequencing should be currently regarded as a reference method or gold standard as it is superior to other methods, including DNA hybrid capture-based sequencing [14]. Therefore, RNA-based testing has been suggested as the method of choice whenever a sequencing device is available [52]. This approach will probably be the most sensitive and specific testing method. However, given the very high prevalence of lung cancer in Western populations, primary RNA-based NGS testing would require enormous financial and technical efforts. Additionally, RNA-based sequencing is known to depend on tissue preservation and RNA quality. Another limitation and potential pitfall of this technology is the need to consider bioinformatically non-pathogenic alterations, which might be detected by these assays. Although RNA-based sequencing can be combined with standard DNA-based mutational analyses to reduce the required time and tissue, these advanced technologies will not be available everywhere. Based on our data, IHC could reduce the number of necessary sequencings. Moreover, as another possible scenario, IHC screening could be carried out locally and only IHC-positive samples could be submitted to a reference laboratory for subsequent sequencing. With this approach, full genomic information on fusion subtypes would be obtained while reducing the number of unnecessary sequencings. However, the reliability of such an algorithm depends extremely on the sensitivity and specificity of immunohistochemistry. This will require rigorous validation and quality management of IHC as well as specific trainings of pathologists to make sure that all positive cases are appropriately recognized. The number of sequencings could be further reduced by considering standard molecular parameters, such as *EGFR*, *KRAS*, and *BRAF* mutations, and *ALK*, *ROS1*, and *RET* rearrangements. Based on our observations and previously published data, these alterations, which reflect specific molecular subtypes of lung cancer, are mutually exclusive with *NTRK* fusions. Thus, sequencings could be further numerically reduced (without losing true positive cases) if only Trk IHC-positive and *EGFR/KRAS/BRAF/ALK/ROS1/RET*-negative samples (tested by appropriate methods, including IHC and NGS) undergo RNA-based sequencing. We were able to test 141 *EGFR/KRAS/BRAF/ALK/ROS1/RET*-negative samples for Trk IHC (Scenario 1) and found only 43 cases positive (30.5%). Certainly, it should be mentioned that in the case of an *EGFR*-mutant NSCLC with secondary resistance to EGFR TKI therapy, *NTRK* testing should be always considered as *NTRK1* fusions have been described as an escape mechanism against EGFR TKI therapy [35]. As an alternative algorithm, *NTRK* testing could be performed after histological confirmation of NSCLC and, in the case of adenocarcinoma, immunohistochemical exclusion of *ALK* or *ROS1* alterations. This pathway requires highly sensitive and specific IHC for *ALK* and *ROS1*. However, due to the low incidence (together <5%), negative findings for *ALK* and *ROS1* limit the number of cases to be investigated only to a small extent.

Moreover, there are NSCLC subtypes that are very unlikely to harbor *NTRK* fusions, such as sarcomatoid carcinomas. However, we do not recommend excluding patients from *NTRK* testing based on tumor morphology since data on correlation with morphologic subtypes of lung cancer are very limited so far and cases of *NTRK* fusions in non-adenocarcinoma NSCLC have indeed been reported.

Another method for molecular *NTRK* testing is fluorescence in situ hybridization. Although we have only very limited data from a small subset of our cohort, we did not observe any discordant cases. We could also detect all positive samples with this method and did not observe any false positives. FISH is a comparably fast testing approach, which, however, has two major disadvantages when compared to NGS: (i) it does not provide full genomic information, i.e., fusion partner genes and breakpoints, and (ii) it requires additional tissue slides for all three genes to be tested. The latter could be overcome in the future if multiplex FISH probes covering at least two *NTRK* genes were available. With a similar amount of material, RNA-NGS has the advantage that further analyses can be carried out on the extracted material, whereas FISH only allows the one examination. Based on the presented data, FISH is also reliable, and therefore it should be included in the proposed workflow as a second choice in case RNA-based NGS is not available, or does not pass quality controls, or exceeds the financial resources.

## 5. Conclusions

In summary, we provide data on the general feasibility of *NTRK* screening on a large series of NSCLC samples under real-world conditions. The prevalence of *NTRK* fusions is 0.2% in unselected lung cancer patients. Our data indicate that a combined approach of Trk IHC pre-screening plus subsequent RNA-based next-generation sequencing is a reliable algorithm to detect patients who would benefit from anti-Trk treatment.

## Figures and Tables

**Table 1:** Compiled overview of previously published cases of *NTRK*-fusion-positive NSCLC.

*NTRK* Gene	Fusion Partner	Histology	Described by
*NTRK1*	*EPS15*	Adenocarcinoma	[14,32], this report
	*SQSTM1*	Adenocarcinoma	[33,34,35], this report
		NSCLC	[14,32,35]
	*TPM3*	Adenocarcinoma	[14,17,21,22,23,24,32,33,35]
	*IRF2BP2*	Adenocarcinoma	[14,21,25,32,33,35]
		Adenocarcinoma with neuroendocrine features	[33]
	*MPRIP*	Adenocarcinoma	[20,33]
	*CD74*	Adenocarcinoma	[20,26,35,36]
	*TPR*	Adenocarcinoma	[32,35]
	*TGF*	Adenocarcinoma	[14]
	*LMNA*	Adenocarcinoma	[35]
	*PHF20*	Sarcomatoid Carcinoma + Adenocarcinoma	[35]
	*BCL9* (intergenic region)	Adenocarcinoma	[35]
	*CLIP1*	Adenocarcinoma	[27]
	*P2RY8*	Adenocarcinoma	[21]
	*GRIPAP1*	Adenocarcinoma	[28]
	*RFWD2*	Large-cell neuroendocrine carcinoma	[29]
	*F11R*	Squamous cell carcinoma	[37]
	*LIPI*	Adenocarcinoma	[38]
*NTRK2*	*STRN*	Adenocarcinoma	[14,23]
	*SQSTM1*	Adenocarcinoma	[17]
	*TRIM24*	Adenocarcinoma	[16]
*NTRK3*	*ETV6*	Adenocarcinoma	[17,24,30,32,33,39]
		Squamous cell carcinoma	[33]
	*SQSTM1*	Adenocarcinoma	[14,23,32,36]
		Neuroendocrine carcinoma	[33]
	*RBPMS*	Adenocarcinoma	[14,23]
	Intergenic region	Large cell neuroendocrine carcinoma	[31]
	*EML4*	Adenocarcinoma	[40]

TPM3, tropomyosin 3; IRF2BP2, interferon regulatory factor 2 binding protein 2; MPRIP, myosin phosphatase rho interacting protein; CD74, cluster of differentiation 74; TPR, translocated promoter region, nuclear basket protein; TGF, transforming growth factor; LMNA, lamin a; PHF20, phd finger protein gene; BCL9, b-cell lymphoma 9; CLIP1, AP-Gly domain containing linker protein 1; P2RY8, P2Y receptor family member 8; GRIPAP1, GRIP1-associated protein 1; RFWD2, also known as COP1 (COP1 E3 ubiquitin ligase); F11R, f11 receptor; LIPI, lipase I; STRN, striatin; TRIM24, tripartite motif containing 24; ETV6, ETS variant transcription factor 6; RBPMS, RNA binding protein; MRNA processing factor; EML4, EMAP like 4.

**Table 2:** Baseline characteristics of cohort.

		Adenocarcinoma		Squamous Cell Carcinoma		Neuroendocrine Tumor		Sarcomatoid Carcinoma		Others ^i^		Total	
Number (*n*, %)		701	71.38 ^ii^	185	18.84	19	1.93	16	1.63	61	6.21	982	
Sex (*n*, %)	Male	408	58.20	133	71.89	11	57.89	10	62.50	36	59.02	598	60.90
	Female	286	40.80	50	27.03	8	42.11	5	31.25	21	34.43	370	37.68
	Unkn.	7	1.00	2	1.08	0	0.00	1	6.25	4	6.56	14	1.43
Age (years) ^iii^	Mean	66.31		67.68		65.78		66.13		69.81		66.73	
	Median	66		68		65.5		68		71		67	
	Range	37–94		36–85		53–92		48–82		48–87		36–92	
	Unkn. (n)	8		3		0		1		4		16	
Molecular alterations (n, %)													
*KRAS* mutation	Pos.	228	32.52	0	0.00	1	5.26	5	31.25	2	3.28	236 ^iv^	24.03
	Neg.	284	40.51	49	26.49	6	31.58	10	62.50	37	60.66	386	39.31
	Unkn.	189	26.96	136	73.51	12	63.16	1	6.25	22	36.07	360	36.66
*EGFR* mutation	Pos.	59	8.42	0	0.00	0	0.00	0	0.00	1	1.64	60 ^v^	6.11
	Neg.	538	76.75	60	32.43	9	47.37	16	100	48	78.69	671	68.33
	Unkn.	104	14.84	125	67.57	10	52.63	0	0.00	12	19.67	251	25.56
*BRAF* mutation ^vi^	Pos.	11	1.57	0	0.00	0	0.00	0	0.00	0	0.00	23	2.34
	Neg.	513	73.18	89	48.11	11	57.89	16	100	43	70.49	660	67.21
	Unkn.	177	25.25	96	51.89	8	42.11	0	0.00	18	29.51	299	30.45
*ALK* fusion	Pos.	16	2.28	0	0.00	0	0.00	0	0.00	0	0.00	16	1.63
	Neg.	604	86.16	56	30.27	12	63.16	15	93.75	50	81.97	737	75.05
	Unkn.	81	11.55	129	69.73	7	36.84	1	6.25	11	18.03	229	23.32
*ROS1* fusion	Pos.	4	0.57	0	0.00	0	0.00	0	0.00	0	0.00	4	0.41
	Neg.	604	86.16	51	27.57	12	63.16	15	93.75	50	81.97	732	74.54
	Unkn.	93	13.27	134	72.43	7	36.84	1	6.25	11	18.03	246	25.05
*RET* fusion	Pos.	4	0.57	0	0.00	0	0.00	0	0.00	0	0.00	4	0.41
	Neg.	340	48.50	39	21.08	8	42.11	12	75.00	30	49.18	429	43.69
	Unkn.	357	50.93	146	78.92	11	57.89	4	25.00	31	50.82	549	55.91
Other ^vii^	Pos.	96	13.69	14	7.57	3	15.79	8	50.00	10	16.39	131	13.34
	Neg.	412	58.77	63	34.05	7	36.84	8	50.00	30	49.18	520	52.95
	Unkn.	193	27.53	108	58.38	9	47.37	0	0.00	21	34.43	331	33.71
PD-L1 (TPS) ^viii^	0	173	35.67	46	30.07	9	69.23	1	9.09	13	35.14	242	34.62
(*n*, %)	1–49	153	31.55	69	45.10	3	23.08	1	9.09	13	35.14	239	34.19
	50–100	159	32.78	38	24.84	1	7.69	9	81.82	11	29.73	218	31.19
	Unkn.	216	30.81	32	17.30	6	31.58	5	31.25	24	39.34	283	28.82

^i^: “Others” include unspecified NSCLC (*n* = 25), NSCLC, NOS (not otherwise specified) (*n* = 23), adenosquamous carcinoma (*n* = 5), large-cell carcinoma (*n* = 3), clear-cell carcinoma (*n* = 1), undifferentiated carcinoma (*n* = 1), adenoid cystic carcinoma (*n* = 1), carcinosarcoma (*n* = 1), and giant-cell carcinoma (*n* = 1); ^ii^: numbers in gray are respective percentages; ^iii^: refers to age at the time of molecular diagnostics; ^iv^: *n* = 93 with G12C mutation; ^v^: Exon 18: *n* = 2; Exon 19: *n* = 31; Exon 20: *n* = 7; Exon 21: *n* = 14; Exon 18 + 20: *n* = 2; Exon 18 + 21: *n* = 2; Exon 19 + 20: *n* = 1; Exon 20 + 21: *n* = 1; ^vi^: all *V600E* mutations; ^vii^: panel including *NRAS* (*neuroblastoma ras proto-oncogene*), *AXL* (*axl receptor tyrosine kinase*), *CTNNB1* (*catenin beta 1*), *DDR2* (*discoidin domain receptor tyrosine kinase 2*), *ERBB2* (*erb-B2 receptor tyrosine kinase 2*), *FGFR2* (*fibroblastic growth factor receptor 2)*, *MAP2K1 (mitogen-activated protein kinase kinase 1*), *MET* (*met proto-oncogene*), *MYB* (*myb proto-oncogene*), *NFE2L2* (*nuclear factor-erythroid 2-related factor 2*), *PIK3CA* (*phosphatidylinositol-4,5-bisphosphate 3-kinase catalytic subunit alpha*), and *PTEN* (*phosphatase and tensin homolog*); ^viii^: expression of PD-L1 (programmed death ligand 1) described by tumor proportion score (TPS) in %.

**Table 3:** Results of NTRK testing.

		Adenocarcinoma		Squamous Cell Carcinoma		Neuroendocrine Tumor		Sarcomatoid Carcinoma		Others		Total	
PanTrk IHC	Pos.	81	11.55	31	16.76	3	15.79	10	62.50	8	13.11	133	13.54
(*n*, %)	Neg.	547	78.03	150	81.08	16	84.21	5	31.25	49	80.33	767	78.11
	Unkn.	73	10.41	4	2.16	0	0.00	1	6.25	4	6.56	82	8.35
FISH *NTRK1*	Pos.	2	0.29	0	0.00	0	0.00	0	0.00	0	0.00	2	0.20
(*n*, %)	Neg.	113	16.12	32	17.30	2	10.53	5	31.25	7	11.48	159	16.19
	Unkn.	586	83.59	153	82.70	17	89.47	11	68.75	54	88.52	821	83.60
*NTRK2*	Pos.	0	0.00	0	0.00	0	0.00	0	0.00	0	0.00	0	0.00
	Neg.	90	12.84	32	17.30	2	10.53	4	25.00	6	9.84	134	13.65
	Unkn.	611	87.16	153	82.70	17	89.47	12	75.00	55	90.16	848	86.35
*NTRK3*	Pos.	0	0.00	0	0.00	0	0.00	0	0.00	0	0.00	0	0.00
	Neg.	87	12.41	30	16.22	2	10.53	4	25.00	6	9.84	129	13.14
	Unkn.	614	87.59	155	83.78	17	89.47	12	75.00	55	90.16	853	86.86
RNA-NGS ^ix^ (*n*, %)	Pos.	2	0.29	0	0.00	0	0.00	0	0.00	0	0.00	2	0.20
	Neg.	124	17.69	43	23.24	4	21.05	10	62.50	11	18.03	192	19.55
	Unkn.	575	82.03	142	76.76	15	78.95	6	37.50	50	81.97	788	80.24

^ix^: Panel including *NTRK1*, *NTRK2*, and *NTRK3*.

## Data Availability

The data presented in this study are available on request from the corresponding author. The data are not publicly available due to patient confidentiality concerns.
